# Heat Shock Protein 70 Serum Levels Differ Significantly in Patients with Chronic Hepatitis, Liver Cirrhosis, and Hepatocellular Carcinoma

**DOI:** 10.3389/fimmu.2014.00307

**Published:** 2014-07-01

**Authors:** Mathias Gehrmann, Melchiorre Cervello, Giuseppe Montalto, Francesco Cappello, Alessandro Gulino, Clemens Knape, Hanno M. Specht, Gabriele Multhoff

**Affiliations:** ^1^Department of Radiation Oncology, Klinikum rechts der Isar, Technische Universität München, Munich, Germany; ^2^Institute of Biomedicine and Molecular Immunology “Alberto Monroy”, National Research Council, Palermo, Italy; ^3^Biomedical Department of Internal Medicine and Specialties, University of Palermo, Palermo, Italy; ^4^Section of Human Anatomy, Department of Experimental Biomedicine and Clinical Neurosciences, University of Palermo, Palermo, Italy; ^5^Euro-Mediterranean Institute of Science and Technology, Palermo, Italy; ^6^Tumor Immunology Unit, Department of Health Science, Human Pathology Section, School of Medicine, University of Palermo, Palermo, Italy; ^7^Clinical Cooperation Group (CCG) – Innate Immunity in Tumor Biology, Helmholtz Centre Munich, German Research Centre for Environmental Health, Munich, Germany

**Keywords:** HCC, serum HSP70, prognostic biomarker, chronic hepatitis, inflammation, liver cirrhosis

## Abstract

Members of the heat shock protein 70 (HSP70) family play an important role in assisting protein folding, preventing protein aggregation and transport of proteins across membranes under physiological conditions. Following environmental (i.e., irradiation, chemotherapy), physiological (i.e., cell growth, differentiation), and pathophysiological (i.e., inflammation, tumorigenesis) stress, the synthesis of heat shock proteins (HSPs) is highly up-regulated, whereas protein synthesis in general is reduced. In contrast to normal cells, many tumor entities including hepatocellular carcinoma (HCC) overexpress HSP70, the major-stress-inducible member of the HSP70 family, present it on their cell surface and secrete it into the extracellular milieu. Herein, the prognostic relevance of serum HSP70 levels in patients with chronic hepatitis (CH; *n* = 50), liver cirrhosis (LC; *n* = 46), and HCC (*n* = 47) was analyzed. Similar to other tumor entities, HSP70 is also present on the surface of primary HCC cells. The staining intensity of intracellular HSP70 in HCC tissue is stronger compared to control and cirrhotic liver sections. HSP70 serum levels in all HCC patients were significantly higher compared to a control group without liver disease (*n* = 40). No significant age- and gender-related differences in HSP70 serum levels were observed in male and female healthy human volunteers (*n* = 86). Patients with CH (*n* = 50) revealed significantly higher HSP70 serum levels compared to the control group, however, these values were significantly lower than those of HCC patients (*n* = 47). Furthermore, a subgroup of patients with LC who subsequently developed HCC (LC-HCC, *n* = 13) revealed higher HSP70 serum levels than patients with LC (*n* = 46, *p* = 0.05). These data indicate that serum HSP70 levels are consecutively increased in patients with CH, LC and liver carcinomas and thus might have a prognostic value.

## Introduction

The incidence of hepatocellular carcinoma (HCC) is increasing dramatically in the Western societies in the last years and HCC is the third leading cause of cancer-related deaths (1). Heavy alcohol intake, tobacco, vinyl chloride, and aflatoxin-B1 toxin can initiate HCC in humans. Apart from toxins, HCC can also arise from a dysregulated expression of small non-coding microRNAs (i.e., miR-122), diabetes, non-alcoholic fatty liver disease, hemochromatosis, liver cirrhosis (LC), and chronic hepatitis (CH) B/C viral infections. The exact molecular mechanisms that promote the transition of diseased liver cells into neoplastic lesions remain to be unsolved. The production of pro-inflammatory cytokines and chemokines, which induces a chronic inflammation in the liver are discussed to increase the risk for a malignant transformation (2–4). These data indicate that a multitude of different parameters including toxins, diseases, and the microenvironment of the host can play a role in the development of HCC (5).

At present, the histological evaluation of liver biopsies using the Edmondson–Steiner classification is the gold standard for the grading of HCC. For patients suffering from HCC with an underlying LC the Barcelona Clinic Liver Cancer group (BCLC) classification is used to describe the tumor volume, the grade of cirrhosis and the patient performance status. Apart from morphological inspections, antibodies directed against cyclase-associated protein 2 (CAP2) or glypican-3 are applied in immunohistochemistry to distinguish different tumor stages and to separate malignant from non-malignant lesions (6). Several other tumor biomarkers, such as p53, mammalian target of rapamycin (mTOR), c-MET, insulin-like growth factor 1 receptor (IGF-1R), histone MacroH2A1 (7), and heat shock proteins (HSP) (8) including HSP70 are frequently up-regulated in tumor biopsies of HCC patients. However, the prognostic value of any of these markers alone is limited since its reliability can be impacted by gender and disease related parameters. Elevated mRNA levels of p53 are not only detected in male tumor patients with undifferentiated tumor stages but also in patients with cirrhosis (5). Similar results were found for an increased expression of mTOR that is also associated with other malignancies (5).

A major disadvantage of biopsy-based biomarkers is their limited availability and the risk to develop infections by the surgical intervention. Soluble, blood-derived biomarkers are superior to biopsies since they are easily accessible and can be taken repeatedly by using minimal invasive methods. In the present study, we aim to evaluate the prognostic significance of the major stress-inducible HSP70 in the serum of patients as a potential biomarker to distinguish patients with chronic inflammation (i.e., patients with CH) and LC from patients with HCC.

Members of the HSP70 family play a pivotal role in assisting protein folding, preventing protein aggregation and transport of proteins across membranes under physiological conditions. Following environmental (i.e., irradiation, chemotherapy, oxygen radicals), physiological (i.e., cell growth, differentiation), and pathophysiological (i.e., inflammation, tumor growth) stress, the synthesis of HSPs in general, but especially that of HSP70, is highly up-regulated, whereas that of other proteins is down-regulated. In contrast to normal cells, tumor cells frequently overexpress HSP70 in the cytosol (9), present it on their plasma membrane (10) and can actively secrete it in lipid vesicles such as exosomes (8, 11). The vesicular export of HSP70 in extracellular fluids was reported to stimulate effector mechanisms of the immune system (11, 12). Immunohistochemical analysis of tumor biopsies suggests that HSP70 in combination with other markers such as glutamine synthetase could serve as a putative diagnostic marker in HCC (6). Apart from HCC (13), an elevated expression of HSP70 was also found in patients with early-stage pancreatic cancer (14). High cytosolic HSP70 levels can promote tumor growth, prevent apoptotic cell death and thus are often associated resistance to therapy and poor prognosis in many different cancer types (8).

## Materials and Methods

### Healthy human volunteers and patients

Eighty-six male and female healthy human volunteers (HEALTHY, *n* = 86) at different ages were enrolled into the study as well as patients suffering from CH (CH, *n* = 50), LC (*n* = 46), HCC (HCC, *n* = 47), and patients without a liver disease (*n* = 40) (Table 2). Approval was obtained by the Ethics Committees of the University Palermo, Italy, and the Klinikum rechts der Isar, Technische Universität München, Germany. All procedures were in accordance with the ethical standards of the responsible institutional and national committees on human experimentation and with the Helsinki Declaration of 1975 as revised in 2008.

Serum samples were collected from human patients with and without liver disease and healthy human volunteers at different ages. Blood samples of patients were taken after overnight fasting. After centrifugation (10 min, 750 × *g*, room temperature), part of the serum was used to assay the main parameters of liver function by routine methods. Serum aliquots of 100–500 μl were stored at −80°C for the measurements of HSP70. Sera were thawed only once for testing. Serological testing for anti-HCV was performed using a commercial third-generation enzyme-linked immunosorbent assay (ELISA) (Ortho Diagnostic System, Raritan, NJ, USA), in accordance with the manufacturer’s instructions. Serum levels of HCV-RNA were evaluated qualitatively by the Amplicor HCV test, version 2.0 (Roche Diagnostics, Basel, Switzerland) and quantitatively at baseline by the Cobas Monitor Test, version 2.0 (Roche Diagnostics). Markers of HBV were tested using the Abbott radioimmunoassay kit (Abbott Laboratories, Abott Park, IL, USA).

### Tumor biopsies

Liver biopsy samples were obtained percutaneously according to the Menghini technique using needles of 1.0 ± 1.2 mm diameter (Surecut, Hospital Service, Rome, Italy). In some cases, HCC was diagnosed using a thin needle (20 Gage, Surecut) under ultrascan control, using a Toshiba SSA 240A apparatus with a 3.5-MHz probe. Tissues from HCC and adjacent liver were obtained from patients undergoing surgical resection. Histologically normal liver tissue was obtained from patients during surgery for cholelithiasis. Written informed consent was obtained in all cases; the protocol was approved by the local Ethics Committee 1(see above). Biopsies in the size range of a few mm were taken during tumor excision.

### Flow cytometry

Single cells from freshly isolated tumor biopsies were prepared by mechanical disruption, as described previously (15). 1 × 10^5^ cells were washed once with 10% FCS in phosphate buffered saline (PBS) and incubated with a FITC-conjugated mouse monoclonal antibody specific for membrane-bound HSP70 (cmHSP70.1, IgG1, multimmune GmbH, Munich, Germany) (16) or a FITC-labeled isotype-matched IgG1 negative control antibody (345815, BD Biosciences, Franklin Lakes, NJ, USA) on ice in the dark for 30 min. After washing, propidium iodide was added and viable cells were immediately analyzed on a FACSCalibur flow cytometer (BD Biosciences). The percentage of cells stained with an isotype-matched control antibody was subtracted from the percentage of cmHSP70.1 antibody positive cells.

### Immunohistochemical staining

Immunohistochemical investigation was performed on specimens fixed in formalin and embedded in paraffin. Four micrometer-thick sections were cut, dewaxed, and hydrated, heated in a microwave oven (three to four cycles of 5 min each) in 10 mM citrate buffer (pH 6.0), then washed twice with PBS for 5 min. All sections were incubated in 3% hydrogen peroxide (v/v) in methanol for 5 min. Immunohistochemistry was performed with the Streptavidin–biotin complex (StreptABC) using rabbit polyclonal antibody against HSP70 (Santa Cruz Biotechnology, Inc., Heidelberg, Germany) at a dilution of 1:200 for 2 h at 37°C. Sections were then incubated for 30 min at RT with biotinylated anti-rabbit immunoglobulin diluted in PBS, with StreptABC for 30 min at RT, and the color was developed with 3-amino-9-ethyl-carbazole (AEC) (Dako, Copenhagen, Denmark) for 5–10 min at RT, and counterstained with Mayer hematoxylin for 3 min. Results were assessed semiquantitatively in blind by three expert pathologists and by counting the proportion of positively stained cells in 10 random high power fields at a 10 and 40× magnification.

### ELISA assays

Total HSP70 levels in serum samples of humans were measured using an HSP70 immunoassay (Duoset, DYC1663, R&D Systems, Minneapolis, MN, USA), according to the manufacturer’s instructions with modified buffers. The ELISA is designed to detect inducible human HSP70. All serum samples were tested in three independent ELISA experiments in duplicates. HSP70 was detected by incubation with HRP-conjugated anti-human Ig followed by HRP-substrate staining (DY999, R&D Systems, Minneapolis, MN, USA). Signals were determined by measuring the absorption at 450 nm in a standard ELISA reader (BioTek, Winooski, VT, USA) with a correction wavelength set to 540 nm.

### Statistical analysis

Statistical analysis was performed using SigmaPlot software delivered by Systat Software, Inc. (San Jose, CA, USA). Results of the levels of soluble HSP70 are presented as standard box plots with boundaries indicating the 25th and the 75th percentile. The line inside boxes indicates the median and the whiskers indicate the 10th and 90th percentile, respectively. All outliers are shown. For comparison between groups of data the Student’s *t*-test or the Mann–Whitney Rank Sum Test were used to evaluate differences. *p*-Values <0.05 are considered to be statistically significant.

## Results

### HSP70 membrane phenotype on biopsies of patients with HCC

**Figure 1:**
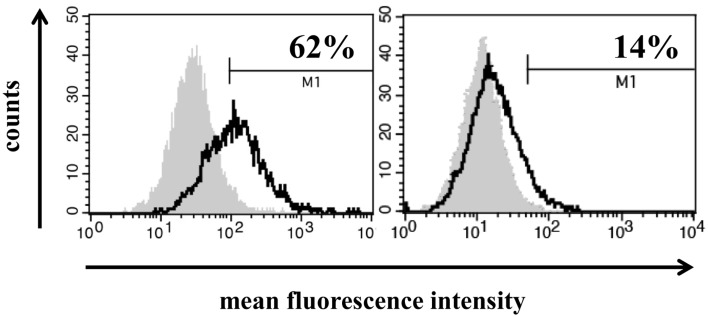
**Representative images of a high (left) and low (right) HSP70 membrane expression on primary HCC cells**. Single cell suspensions from freshly isolated HCC biopsies derived from two different patients were incubated with FITC-conjugated mouse monoclonal antibody cmHSP70.1 that recognizes the membrane-bound form of HSP70 on the surface of tumor cells. The white histogram represents the HSP70 membrane staining of the tumor cells and the gray histogram the staining with a negative control FITC-conjugated isotype-matched control antibody. The percentage of HSP70 membrane-positive cells was corrected by subtraction of the isotype control.

Freshly isolated, non-fixed biopsies of patients with HCC were minced and filtered through a sterile mesh to obtain single cell suspensions of the tumor. Directly after washing tumor cells were centrifuged and incubated with FITC-conjugated mouse monoclonal antibody (mAb) cmHSP70.1 that recognizes the membrane-bound form of HSP70 on the surface of viable tumor cells with an intact membrane (Figure 1, white histograms). As a negative control, a FITC-conjugated isotype-matched control antibody was used (Figure 1, gray histograms). The percentage of HSP70 positively stained cells was corrected according to the results from the staining with an isotype-matched control antibody. The histograms depicted in Figure 1 show a typical result of a high (62%) and low (14%) membrane HSP70 positive tumor biopsy. Previous studies with biopsies of normal tissues revealed that a sample is considered as HSP70 membrane-positive if more than 20% of the cells are stained positively with the cmHSP70.1 mAb (17). With respect to this threshold, three out of five selected HCC patient samples were HSP70 membrane-positive (Table 1). This finding is in line with results derived from other human tumor entities (*n* = 978), showing that more than 50% of all tested tumor samples were found to be HSP70 membrane-positive (10, 15).

**Table 1:** **HSP70 membrane status in single cell suspensions of HCC tissue obtained from tumor patients**.

Patient no.	HSP70^+^ cells (%)	Mean fluorescence intensity (MFI)	HSP70 phenotype
1	62	429	+
2	95	1923	+
3	33	158	+
4	12	27	−
5	14	37	−

### Representative immunohistochemical analysis of the HSP70 protein content in liver sections of human volunteers without liver disease (CTRL) and HCC patients with underlying LC

The patient cohort consists of 183 subjects that could be divided into four groups (Table 2). Group 1 was composed of 40 patients (CTRL) without liver disease derived from the Biomedical Department of Internal Medicine and Specialties, University of Palermo, Palermo, Italy. Liver disease was excluded on the basis of anamnestic, biochemical, and instrumental data. In this group, no case of neoplastic disease was detected within a follow-up period of at least 6 months. Group 2 included 50 patients with CH infections and Group 3 included 46 patients with LC. Diagnosis was made on the basis of liver biopsies and unequivocal biochemical and instrumental data. The absence of neoplasia had been verified during a post-study follow-up period of at least 6 months. Finally, group 4 included 47 patients with HCC. Diagnosis was based on histology, cytology, multiple concordant imaging techniques [ultrasound, basal and lipiodol computed tomography (CT), selective angiography], and biochemical assays (serum levels of AFP >200 ng/ml). Some of the patients were known as liver cirrhotics and had been enrolled in a prospective study for HCC screening.

**Table 2:** **Clinical and pathological features of control patients without liver diseases (CTRL) and patients with chronic hepatitis (CH), liver cirrhosis (LC), and hepatocellular carcinoma (HCC); data are expressed as the median (range)**.

Characteristics	Group 1 CTRL	Group 2 CH	Group 3 LC	Group 4 HCC
Number (*n*)	40	50	46	47
Gender (M/F)	36/4	30/20	24/22	27/20
Age (years)	44 (23–63)	52.5 (25–85)	66.5 (30–86)	73 (45–87)
Albumin (g/dl)	4.7 (3.47–5.01)	4.66 (4.0–4.9)	3.4 (2.0–4.5)	1.03 (0.24–5.56)
Bilirubin (mg/dl) total	0.72 (0.52–1.0)	0.75 (0.34–1.1)	1.27 (0.15–5.89)	1.03 (0.24–5.56)
Aspartate amino-transferase (IU/ml)	18.7 (12.0–26.1)	55 (25.0–173.0)	70 (19.0–377.0)	56 (12.0–204.0)
Alanine amino-transferase (IU/ml)	16.2 (11.5–22.02)	85 (31.0–251.0)	57 (12.0–221.0)	45 (12.0–230.0)
International normalized ratio (INR)	0.92 (0.86–1.01)	0.97 (0.91–1.07)	1.24 (1.06–1.73)	1.06 (0.82–1.75)
HBs Ag	–	2	4	3
HCV Ab	–	38	32	34
Alcoholism	–	None	3	5
Cryptogenic	–	10	7	4
Dysmetabolic	–	None	None	1
**BCLC**				
A	–	–	–	21
B	–	–	–	9
C	–	–	–	8
D	–	–	–	5
E	–	–	–	4
**CHILD-PUGH**				
A	–	–	26	–
B	–	–	16	–
C	–	–	4	–

*BCLC, Barcelona Clinic Liver Cancer group; HBs Ag, anti-hepatitis B surface antigen; HCV Ab, anti-hepatitis antibody*.

**Figure 2:**
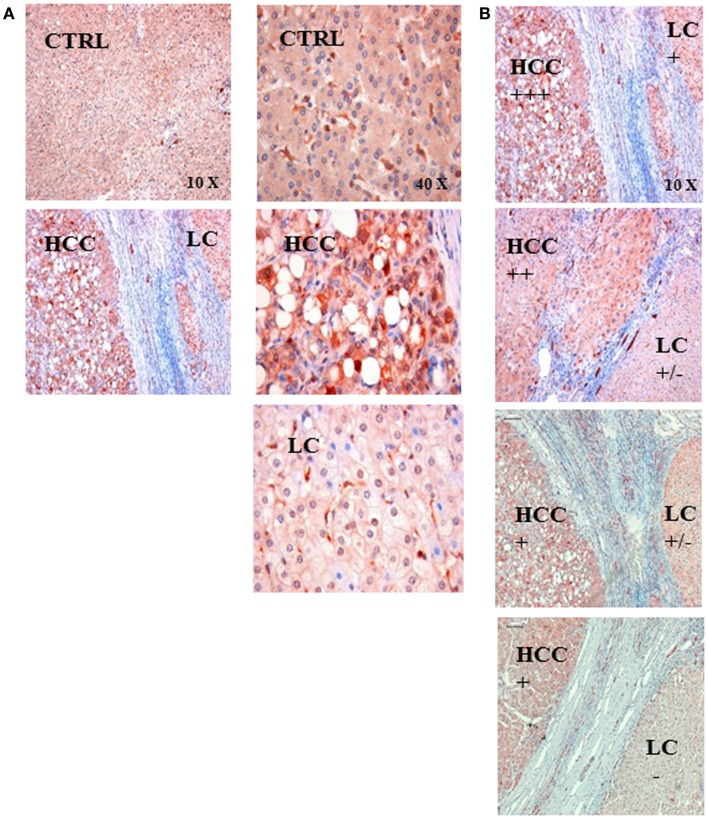
**(A)** Representative immunohistochemical images of the HSP70 staining in control liver tissue (CTRL) and in the tissue of a patient with hepatocellular carcinoma (HCC) with an underlying liver cirrhosis (LC). The HSP70 staining intensity was stronger in HCC tissue compared to that of control liver tissue (CTRL) and in areas with liver cirrhosis (LC); left panel 10× magnification, right panel 40× magnification. **(B)** Semiquantitative analysis of the HSP70 staining intensity in sections of LC-HCC patients (*n* = 4) at a 10× magnification. The HSP70 staining intensity in the HCC regions ranged from very strong (+++), via intermediate (++) to strong (+); in the LC regions the staining intensity ranged between strong (+), weak (±), and very weak (−) in the four different sections.

Representative immunohistochemical images were taken from sections of liver biopsies of human patients without liver disease (CTRL) and a patient who was diagnosed with HCC and LC (Figure 2A). The HSP70 staining intensity was stronger in the HCC tissue compared to that of the control liver (CTRL) and to the cirrhotic part (LC) of the patient biopsy. These data indicate that the intracellular HSP70 content is higher in the cancerous compared to the cirrhotic liver tissue. A comparison of four different LC-HCC patients revealed different staining intensities in the HCC and LC regions and between the different patient sections (Figure 2B).

### HSP70 protein levels in the serum of human volunteers (healthy), patients without liver disease (CTRL), and patients with chronic hepatitis, liver cirrhosis, and hepatocellular carcinomas

**Figure 3:**
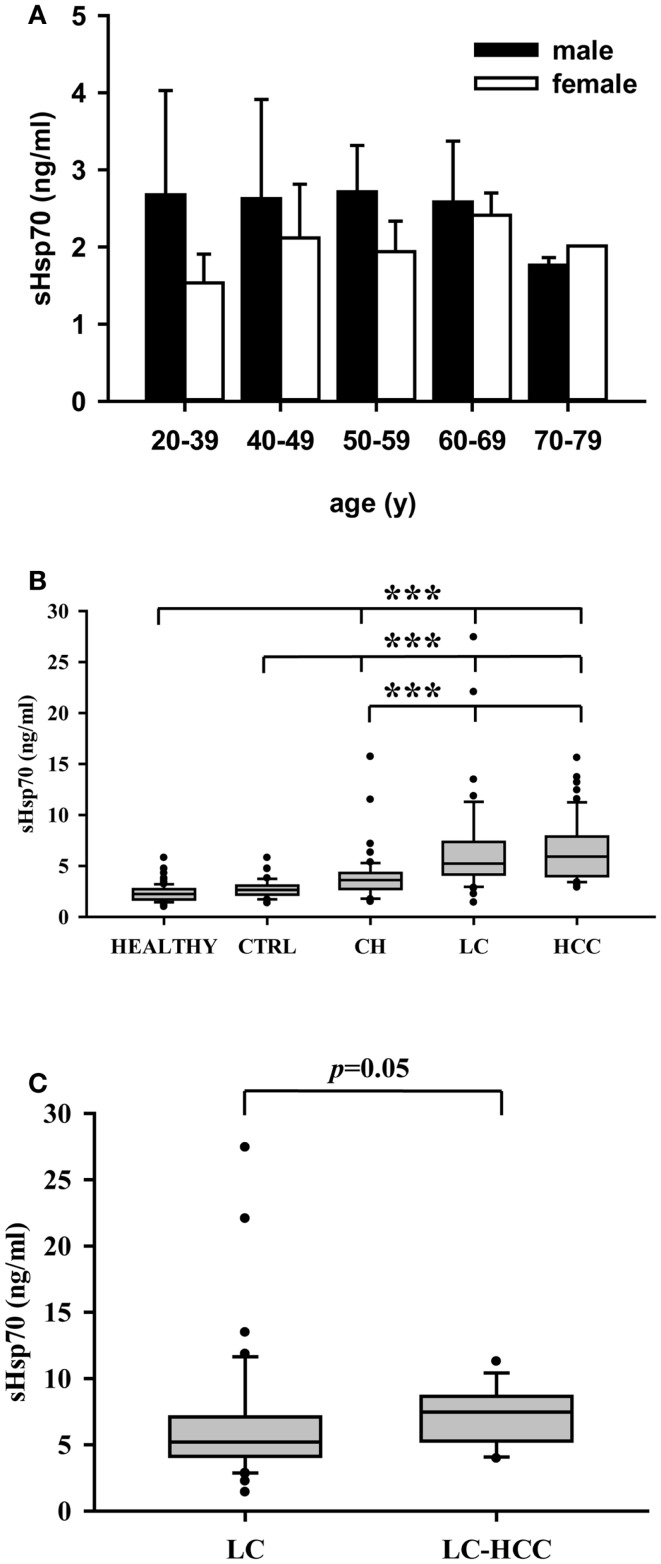
**(A)** HSP70 protein levels in the serum of male (*n* = 54) and female (*n* = 32) healthy human volunteers at different ages (*n* = 86). The HSP70 serum levels did neither differ significantly in male and female healthy human individuals nor in different age groups ranging from 20–39, 40–49, 50–59, 60–69, to 70–79. **(B)** HSP70 protein levels in healthy human volunteers (HEALTHY, *n* = 86), healthy controls without liver diseases (CTRL, *n* = 40), patients with chronic hepatitis (CH, *n* = 50), liver cirrhosis (LC, *n* = 46), and hepatocellular carcinomas (HCC, *n* = 47). Serum HSP70 levels did not differ significantly between healthy human volunteers and controls without liver diseases, but both groups differed significantly to patients with CH, LC, and HCC. **(C)** HSP70 protein levels in the serum of patients with liver cirrhosis (LC, *n* = 46) and of HCC patients with an underlying LC (LC–HCC, *n* = 13). Serum HSP70 levels were higher (*p* = 0.05) in patients with HCC and an underlying LC compared to patients with LC only. Serum HSP70 levels were determined by sandwich ELISA. Values were determined at least three times in duplicates. Median values are shown as box plots. Significance was calculated by using the Mann–Whitney-*U*-test (***p  < 0.001).

The soluble HSP70 levels were determined in the serum of 86 male (54) and female (32) healthy human volunteers at different ages. Irrespectively of the age and gender, the HSP70 serum levels did not differ significantly in the healthy human volunteers (HEALTHY) (Figure 3A). A comparison of the HSP70 levels in patients without liver disease (CTRL, *n* = 40, 2.7 ± 0.9 ng/ml) and healthy human volunteers (HEALTHY, *n* = 86, 2.3 ± 0.8 ng/ml) also revealed no significant differences (Figure 3B). In contrast, the HSP70 serum levels of patients with liver diseases such as CH, LC, and HCC differed significantly from that of healthy human volunteers and patients without liver disease. The highest serum HSP70 levels in patients were found in HCC (HCC, *n* = 47, 6.5 ± 3.1 ng/ml) and LC patients (LC, *n* = 46, 6.6 ± 5.2 ng/ml). The lowest HSP70 levels were found in patients with CH (*n* = 50, 3.9 ± 2.4 ng/ml). These values were significantly lower than that of HCC and LC patients (Figure 3B).

In order to evaluate whether the HSP70 serum levels could predict the risk to develop HCC a subgroup analysis was performed. As shown in Figure 3C, a small subgroup of HCC patients with underlying LC revealed higher HSP70 serum levels (LC-HCC, *n* = 13, 7.3 ± 2.2 ng/ml) than the overall group of patients with LC (6.6 ± 5.2 ng/ml) with unknown HCC status. However, due to the low number of patients (*n* = 13) only a trend (*p * = 0.05) was determined.

## Discussion

Biomarkers are used to detect tumors, monitor tumor growth, and to assess the effectiveness of anti-cancer therapies (18). A major disadvantage of biopsy-based markers is the risk for developing infections caused by the invasive intervention. Since blood samples can be taken by minimal invasive methods from patients before, during, and after therapy this method is superior for tumor detection and for monitoring the clinical outcome. In this study, soluble HSP70 was examined for its potential prognostic significance to serve as a blood-derived biomarker to detect HCC and to distinguish HCC from other liver diseases such as CH and LC. Previous studies of our group already have shown that HSP70 membrane-positive tumors actively secrete HSP70 into the extracellular milieu in cell cultures (11). This result could be confirmed in tumor bearing mice (19) and in patients with squamous cell carcinomas of the head and neck (Ms submitted). Since the availability of tumor biopsy material is limited during the course of disease, we addressed the question whether serum HSP70 levels could reflect the HSP70 membrane status of the tumor cells also in HCC patients. Comparative analysis revealed that an increased intracellular HSP70 staining intensity of HCC cells in sections was associated with increased serum HSP70 levels in a selected group of patients who suffered from HCC and LC (data not shown). In a group of patients with HCC only, the cytosolic HSP70 levels did not correlate with soluble HSP70 levels. This is in line with the findings of Kang et al. (5) who showed no correlation of cytosolic HSP70 levels with prognosis of HCC after resection. The excellent accessibility of serum biomarkers allows repeated testing during the course of a disease and the monitoring of clinical outcome. Serum HSP70 levels have been discussed to provide a useful biomarker for testing the efficacy of an Hsp90 inhibitor-based tumor therapy that is known to induce the expression of HSP70 (20).

It has been reported that HSP70 can be actively released by viable tumor cells with an intact cell membrane (21). In this study, we could show that patients with HCC exhibited significantly higher HSP70 serum levels compared to patients with hepatic viral infections (Figure 3). These findings might provide a hint that the largest proportion of soluble HSP70 in the serum is produced by viable tumor cells that actively secrete HSP70 in lipid vesicles and not by necrosis of inflamed liver tissue. Together with the finding that serum HSP70 levels correlate with the volume of viable tumor cells in mice (19), we hypothesize that soluble HSP70 levels might be useful to evaluate the mass of vital tumor cells in human patients before and after therapeutic intervention.

Since membrane HSP70 is frequently present on a broad variety of different tumor entities such as colorectal, lung, pancreatic, and prostate cancer patients (14, 20, 22, 23) and since membrane HSP70 positive tumor cells do secrete HSP70 into the extracellular milieu it is expected that soluble HSP70 levels might serve as a useful biomarker for different tumor entities. Elevated HSP70 serum levels have been found in cardiovascular, inflammatory and pregnancy-related diseases. In this study, we could show quantitative differences in soluble HSP70 levels in inflammation, cirrhosis, and cancer. Since the highest amount of HSP70 is actively secreted by tumor cells and not from inflamed and virally infected tissues, soluble HSP70 levels might provide a measure to determine the mass of viable tumor cells in patients (24).

## Conclusion

In the present study, the prognostic value of extracellular HSP70 was determined in the serum of patients with liver diseases such as CH, LC, and HCC. HSP70 serum levels were found to be significantly higher in cancer patients compared to healthy individuals, patients without liver diseases and patients with an inflammation of the liver. Our data encourage us to hypothesize that serum HSP70 might be a useful biomarker to differentiate HCC from other liver diseases.

## Conflict of Interest Statement

The authors declare that the research was conducted in the absence of any commercial or financial relationships that could be construed as a potential conflict of interest.
